# Enhancing FT-MIR Prediction of Short-Chain Free Fatty Acids in Milk Through High-Lipolysis Field Samples, Reference Method Evaluation and Machine Learning Approaches

**DOI:** 10.3390/foods15193423

**Published:** 2026-09-24

**Authors:** Octave S. Christophe, Didier Veselko, Julie Leblois, Frédéric Dehareng, Hélène Soyeurt

**Affiliations:** 1 Walloon Agricultural Research Center (CRA-W), 24 Chaussée de Namur, 5030 Gembloux, Belgium; 2 Gembloux Agro-Bio Tech, TERRA Teaching and Research Centre, University of Liège, 5030 Gembloux, Belgium; hsoyeurt@uliege.be; 3 Comité du Lait de Battice, Route de Herve 104, 4651 Battice, Belgium; didier.veselko@cdl-battice.be; 4 Elevéo Asbl, AWE Group, 4, Rue des Champs Elysées, 5590 Ciney, Belgium; jleblois@awegroupe.be

**Keywords:** milk, mid-infrared spectroscopy, machine learning, lipolysis, free fatty acids

## Abstract

Lipolysis is a longstanding and appealing topic within the dairy industry. Having been extensively researched throughout the twentieth century, lipolysis is often prematurely dismissed as a settled or outdated field of study. In recent years, the advent of robotic milking systems and increased milking frequencies have led to a rise in lipolysis levels, sparking renewed interest in the monitoring of milk quality. Predicting lipolysis more effectively relies on the analysis of free fatty acid (FFA) concentrations. Recent research has demonstrated the feasibility of quantifying individual FFA in raw milk. In particular, Fourier-transform mid-infrared (FT-MIR) spectroscopy has shown considerable potential for predicting these compounds. However, that study presented several limitations, including the use of non-standardized reference methods and the reliance on artificially lipolyzed samples to enhance variability within the calibration dataset. To overcome these limitations, FT-MIR FFA models have been used as a preliminary screening tool in order to select samples (n = 248) with high lipolysis, and the selected samples were analyzed using the ISO reference method. A total of 248 samples were selected, of which 208 were obtained through the Walloon DHI in Belgium and 40 were sampled at the CRA-W experimental farm. A comparison between the two reference methods was performed and highlighted the need to harmonize reference analysis as the two reference methods are only comparable for short-chain free fatty acids (SCFFA). A mixed model for SCFFA combining both references was built using different algorithms such as Partial Least Squares (PLS), Kernel Ridge Regression (KRR), Support Vector Machine Regression (SVR), Random Forest Regression (RFR) and Gaussian Process Regression (GPR). The model developed for SCFFA showed predictive performances comparable to those reported in the literature with an *R*^2^val of 0.80 and an RMSEval of 32.08 mg/L. Qualitative models for SCFFA were created with a threshold highlighted in the literature using the same algorithms as the quantitative model and gave an accuracy of correct classification of 85% applied on the test dataset. This work has highlighted the necessity to correctly select the reference method in order to build the calibration dataset. Non-linear algorithms provided analyte-dependent improvements over PLS, particularly for C6, C8 and SCFFA prediction.

## 1. Introduction

For decades, milk has been collected for its significant nutritional benefits. It serves as an excellent source of hydration [1] and provides essential dietary proteins [2] and fats [3,4]. The lipid fraction of milk is primarily composed of triacylglycerols (TAG), which account for approximately 98% of its total fat content.

In the human body, these TAG serve as a major energy reservoir through the release of fatty acids. Interestingly, a similar degradative process can occur within the milk itself. This biochemical breakdown is catalyzed by the enzyme lipoprotein lipase (LPL), which is naturally indigenous to milk.

This enzyme exhibits substantial lipolytic activity, approximately 1 mmol FFA/min/L of milk, meaning that, when sufficient substrate is accessible, it can hydrolyze milk fat, leading to rancidity [5]. Despite this potential, most milk remains stable because the milk fat globule membrane (MFGM) serves as a protective barrier, shielding the triglycerides from enzymatic attack [5]. Within dairy science, lipolysis is generally classified into three distinct categories based on its cause: spontaneous, induced, and microbial [5].

The mechanism underlying spontaneous lipolysis remains incompletely understood, but several factors are known to directly influence the concentration of FFA such as mastitis, high somatic cell count [6], late lactation stage [7], parity, milk production, milking frequency [8,9] and different feeds and feeding systems [10]. These factors have been identified as risk factors for cows to produce milk susceptible to spontaneous lipolysis.

Induced lipolysis results from mechanical stresses that damage the MFGM, thereby allowing LPL to access TAG, which are subsequently hydrolyzed. Disruption of the MFGM and/or the reduction of the fat globule size through a mechanical induction of milk is the best process to enhance milk FFA concentration. Indeed, Christophe et al. [11] demonstrated a strong relationship between different mechanical actions and the increase in FFA concentration. In practical dairy production systems, spontaneous and induced lipolysis frequently coexist and are therefore difficult to distinguish analytically. In contrast, microbial lipolysis is strictly dependent on the microbiological quality of the milk and is catalyzed by exogenous lipases. These enzymes are typically produced by psychrotrophic bacteria during refrigerated storage [5]. Even in raw milk of high initial hygienic quality, the impact of microbial lipases becomes significant after 3 to 4 days of cold storage, potentially compromising the stability and sensory attributes of the final product.

The quantification of lipolysis has remained a significant analytical challenge since the inception of dairy science. Increasingly, the individual profiling of FFA has gained importance, as each fatty acid contributes differently to the sensory characteristics and flavor profile of milk. However, precise measurement is hindered by two primary technical obstacles: the critical nature of sample stabilization and the biochemical complexity of FFA recovery. Because LPL remains active post-milking, immediate stabilization—typically through rapid pasteurization or thermal denaturation—is essential to arrest enzymatic activity and ensure that the FFA levels measured reflect the state of the milk at the time of collection [12]. Furthermore, the subsequent extraction and separation of FFA from the complex milk matrix are technically demanding. The high affinity of fatty acids for milk proteins and the structural interference of the milk fat globule membrane necessitate sophisticated titrimetric or chromatographic methods to achieve accurate recovery and quantification. Due to these inherent challenges in quantifying FFA, various analytical methods have been studied, ranging from methods providing only partial recovery for total FFA quantification to those achieving complete separation and recovery of individual fatty acids.

One of the earliest methods to determine the concentration of FFA was developed by Thomas et al. [13] in 1955. This method consists of dissolving a known amount of fat in a nonionic surfactant/detergent (polyphosphate and Triton X-100). The separated milk fat is then titrated using a standardized base (tetra-n-butylammonium hydroxide solution). The milk fat acidity was expressed as the amount of base required to neutralize the FFA present in 100 g of milk fat (meq/100 g of fat). This method was furthermore standardized by the “Bureau of Dairy Industries” and given the name BDI method. However, a major limitation of this method is that only 50 to 70% of the total FFA are recovered from milk due to its detergent separation and titrimetric quantification approach [14].

Another popular method to quantify total FFA was the copper soap method [15,16]. This quantification is a colorimetric method based on the reaction of the FFA contained in dairy products with copper ions to form a complex. The extract is mixed with a coloring agent (diethyldithiocarbamate). The concentration is then inversely proportional to the intensity of the yellow coloration measured at 440 nm. Despite its age, this methodology remains widely used and continues to undergo refinement [17] due to its operational simplicity and relatively high recovery of total FFA compared to the titrimetric approaches.

These analytical approaches are limited by their focus on the overall quantification of FFA. To achieve a deeper understanding of lipolysis and its specific impact on organoleptic quality, more detailed data are required. For instance, short-chain free fatty acids (SCFFA) are generally recognized as the primary contributors to hydrolytic rancidity and flavor defects in milk and dairy products compared with long-chain FFA [5,18,19].

To distinguish these individual FFA, chromatographic separation is essential. A recently standardized analytical method based on the work of Mannion et al. [20], resulting from an international collaboration between researchers and codified as ISO 24191:2024|IDF 263:2024 [21], is currently emerging as the global reference standard. This methodology was selected over established chromatographic techniques due to several strategic advantages [11,22]. Beyond its versatility across all dairy matrices, the method utilizes butyl ester derivatization to ensure the quantitative recovery of volatile SCFFA that are frequently lost with methyl ester derivatization [20,23]. Furthermore, the integration of a flame ionization detector (FID) provides a robust and cost-effective analytical solution compared to more expensive detection systems, maintaining high sensitivity while remaining accessible for routine laboratory use.

Although chromatographic techniques provide high analytical performance, they are costly, labor-intensive, and unsuitable for large-scale routine testing. In contrast, FT-MIR-based predictive approaches have become widely adopted in the dairy industry over the past decade because of their efficiency and practicality. These models are now commonly used to support and optimize farm management decisions. Indeed, FT-MIR-based prediction models have been extensively developed for traits such as milk components like fatty acids [24], minerals [25] and indirect traits such as methane emitted by cows [26], the nitrogen use efficiency [27] and the chronic stress of dairy cows [28,29]. Christophe et al. [11] have already developed FT-MIR models to predict FFA in milk but that work was mainly composed of induced-lipolysis samples.

The present study was designed to improve the robustness and applicability of FT-MIR models for predicting FFA in milk. First, the agreement between the historical chromatographic method used by Christophe et al. [11] and the newly standardized ISO 24191:2024|IDF 263:2024 reference method was evaluated [21]. Second, the existing calibration dataset was enriched with field samples exhibiting high levels of spontaneous lipolysis in order to increase biological variability and improve model robustness under practical conditions. Third, several linear and non-linear machine learning algorithms were compared for the prediction of SCFFA, which are the primary contributors to lipolysis-related flavor defects in milk. Finally, classification models were developed to identify samples at risk of lipolysis-related sensory defects and to assess their potential as screening tools for routine milk quality monitoring.

## 2. Materials and Methods

### 2.1. Milk Sampling

All samples were collected following the guidelines established by the International Committee for Animal Recording (ICAR, 2022) using ICAR-approved milk sampling devices. The sampling campaign allowed the collection of 241 individual cow samples and 7 bulk milk samples originating from different dairy breeds across Belgium. Of these, 208 samples were obtained via the Association Wallonne de l’Élevage (AWE, Ciney, Belgium) Dairy Herd Improvement (DHI) through the milk recording program, while the remaining 40 samples were collected at the Centre Wallon de Recherches Agronomiques (CRA-W, Gembloux, Belgium) experimental farm. To maximize the variability represented within the calibration dataset, sample selection was strategically guided by the predictive MIR model previously developed by Christophe et al. [11].

In order to address the scarcity of naturally high-lipolysis samples noted in our earlier research, we adopted a targeted sampling approach in partnership with the regional DHI and the Comité du Lait (CDL, Battice, Belgium). The routine FT-MIR predictions derived from the previous SCFFA model served as an initial screening mechanism within the DHI spectral acquisition. For the sake of operational efficiency during sample collection, a screening threshold of 50 mg/L was selected, which closely aligns with the recognized sensory defect threshold of approximately 51.8 mg/L. Samples that surpassed the 50 mg/L threshold were “preferentially selected”, indicating that they were specifically chosen for the expensive and labor-intensive reference chromatographic analysis to enhance the high-concentration tail of the calibration set. In contrast, no samples predicted to be below 50 mg/L were kept from this particular campaign, since the lower concentration range had already been thoroughly represented by the dataset from Christophe et al. [11].

To ensure a high level of FFA concentration, samples were initially screened via FT-MIR spectroscopy by the Comité du Lait (CDL, Battice, Belgium). Samples with predicted SCFFA concentrations exceeding 50 mg/L were preferentially selected for reference analysis. All selected samples underwent rigorous reference analysis (ISO 24191:2024|IDF 263:2024) to determine their definitive FFA profiles.

### 2.2. Chromatographic Analysis

FFA in milk were extracted and analyzed according to ISO 24191:2024|IDF 263:2024, derived from Mannion et al. [20]; minor adaptations were introduced to accommodate laboratory-specific instrumentation while preserving the analytical principles of the method. A 10 mL milk aliquot was transferred into a 50 mL centrifuge tube (Greiner Bio-One, Vilvoorde, Belgium) and mixed with 10 mL of ethanol. An internal standard (ISTD) mixture (1 mL) containing pentanoic acid (C5:0) (Larodan, Solna, Sweden), undecanoic acid (C11:0) (Larodan, Solna, Sweden), and heptadecanoic acid (C17:0) (Larodan, Solna, Sweden), each at a concentration of 1 g/L in heptane, was added to the sample. To induce protein precipitation and lipid separation, 0.3 mL of 2.5 M sulfuric acid was introduced. The lipid fraction was then extracted three times using a diethyl ether/heptane mixture (50:50, *v*/*v*) in successive volumes of 15 mL, 15 mL and 10 mL. After each addition, the tubes were shaken and centrifuged at 3000 rcf for 5 min, and the upper organic phase was collected and transferred to a clean centrifuge tube for subsequent analysis.

The next step was the separation of fat through solid-phase extraction (SPE). The pooled organic extract was subsequently purified using an aminopropyl SPE cartridge (Chromabond, Macherey-Nagel, Düren, Germany) mounted on a vacuum manifold. Throughout the entire extraction process, care was taken to prevent the aminopropyl phase from drying out, applying additional milliliters of heptane between steps when necessary. Prior to sample loading, the cartridge was conditioned with 10 mL of heptane. The extracted lipid fraction was then transferred onto the column and allowed to elute completely. To remove neutral lipids, the cartridge was washed with 10 mL of a hexane/diethyl ether solution (20:80, *v*/*v*). The FFA fraction was then selectively eluted by adding 5 mL of a formic acid/diethyl ether solution (2:98, *v*/*v*), and the eluate was collected in a 10 mL amber screw-cap glass tube.

The next step of the analysis was derivatization: a 600 µL aliquot of the purified FFA extract was transferred into a glass reaction vessel, followed by the addition of 600 µL of the esterification solution: boron trifluoride 10% in 1-butanol solution (LiChropur, Merck, Darmstadt, Germany) and 2.8 mL of heptane. The mixture was vortexed for 20 s and incubated at 80 °C for 1 h to form butyl esters. After cooling to room temperature, a washing step was required to remove most of the excess butanol: 4 mL of a water/methanol solution (1:1) was added, and the mixture was vigorously vortexed for 2 min, taking care to avoid the formation of a persistent emulsion. The upper organic layer containing the fatty acid butyl esters (FABEs) was collected and washed twice, each with 4 mL of the water/methanol solution and 2 min of vigorous vortexing. Finally, a 1 mL aliquot of the washed upper organic layer was transferred into a 2 mL amber screw-cap vial prior to chromatographic injection.

The chromatographic injection was performed with 1 µL of the sample through a split injector heated at 250 °C with a split ratio of 50 in a GC-2010 gas chromatograph (Shimadzu, Kyoto, Japan). The column used was a CP-Sil 88 (CP7489) (Agilent, Santa Clara, CA, USA) of a length of 100 m with an inner diameter of 0.25 mm and a film thickness of 0.2 µm. The column temperature gradient started at 40 °C for 2 min, then increased at a rate of 7.5 °C/min up to 240 °C and the final temperature was maintained for 15 min with a constant flow of helium of 1.2 mL/min. The compounds were then analyzed with an FID at a constant temperature of 280 °C. Peak integration was performed using LabSolutions software (v 5.97 SP1) (Shimadzu, Kyoto, Japan). In order to obtain the retention times and to perform quantification, a standard solution (GLC-74 free acid, Nu-Chek Prep, Elysian, MN, USA) composed of Butyric acid (C4:0), Caproic acid (C6:0), Caprylic acid (C8:0), Capric acid (C10:0), Lauric acid (C12:0), Myristic acid (C14:0), Palmitic acid (C16:0), Stearic acid (C18:0), Oleic acid (C18:1), Linoleic acid (C18:2), Linolenic acid (C18:3), Arachidic acid (C20:0) and Behenic acid (C22:0) was used in this work. Owing to their low concentration in milk, C18:3, C20:0 and C22:0 were not included in the quantitative analysis.

In samples evaluated according to this method, a reported value of 0 mg/L signifies that the concentration of the target analyte was definitively below the method’s Limit of Quantification (LOQ). It is crucial to highlight that the historical dataset approached lower limits in a different manner; within that dataset, the minimum reported thresholds were directly associated with the lowest concentration of the analytical standard utilized for calibration during that period.

### 2.3. Comparison of Data

In this study, two independent datasets generated using different chromatographic methodologies were compared. The first dataset corresponds to the 792 samples described by Christophe et al. [11], including both 313 spontaneous lipolysis samples and 479 induced lipolysis samples. The second dataset comprised the 248 milk samples analyzed in the present study using the ISO 24191:2024|IDF 263:2024 reference method adapted from Mannion et al. [20]. Descriptive statistics were compared to evaluate differences in concentration distributions between datasets.

In addition, 25 independent milk samples covering a wide range of lipolysis levels were analyzed using both analytical methods. Agreement between methods was assessed through linear regression using slope, bias, and coefficient of determination (R^2^).

The comparability of the two reference methods was also statistically assessed using Bland-Altman analysis to quantify mean systematic bias, alongside Deming and Passing-Bablok regressions to evaluate both constant and proportional biases while accounting for measurement errors in both analytical procedures.

Because the objective was to integrate the datasets for FT-MIR model development, evaluating the comparability of both reference methods represented a prerequisite for subsequent analyses.

### 2.4. Milk Spectral Analysis

FT-MIR spectra were acquired using MilkoScan FT7 instruments (Foss, Hillerød, Denmark) at the CDL and CRA-W. The spectra were standardized using the European Milk Recording (EMR) method, as outlined in the study conducted by Grelet et al. [30]. FT-MIR spectra were pre-processed using a first derivative transformation with a gap size of 5 wavenumbers. The selected spectral area consisted of 212 wavenumbers from 968.1 to 1577.5 $cm^{-1}$, 1731.8 to 1762.6 $cm^{-1}$, 1781.9 to 1808.9 $cm^{-1}$, and 2831.0 to 2966.0 $cm^{-1}$ to exclude spectral areas not reproducible between instruments [31] and the absorbance values were mean-centered before being modeled. Each sample underwent triplicate analysis using the FT-MIR spectrometer. Instead of averaging these measurements—which might unintentionally include dilution or cross-contamination from the previous sample—only the third replicate was selected for model development. This approach is a common practice in flow-cell milk spectroscopy, as it guarantees that the instrument’s fluidic system is thoroughly flushed and saturated with the current sample, thus significantly reducing carry-over effects.

### 2.5. Model Development

Five distinct algorithms (machine learning and chemometric) were used for both the quantification of data (regression) and the classification of data into groups or categories (classification). The algorithms that were initially chosen to provide a baseline for the project were Partial Least Squares Regression (PLSR) and PLS-Discriminant Analysis (PLS-DA) as they represent the gold standard in model development in FT-MIR. The PLS algorithm is, in this case, used to determine a new latent space that will maximize the covariance between both the predictor variables and the response variables [32]. The number of latent variables (LVs) was optimized to give the best-fitting model for the data.

The first non-linear algorithm employed in this work is Kernel Ridge Regression (KRR) [33]. KRR is well-suited for medium-sized datasets [34], as it efficiently handles non-linear relationships while maintaining a relatively low computational cost. KRR is used to take Ridge Regression (which consists of linear least squares via L2-norm regularization) and apply the ‘kernel trick.’ By using different kernels, data points can now be mapped into a higher dimensional feature space in order to build models that can illustrate non-linear relationships. The different kernels tested for building the FFA models were linear, radial basis function (RBF) and polynomial. During cross-validation, we optimized the KRR regularization parameter (α) and kernel hyperparameters by performing a grid search over the range $\left[10^{-4},10^{4}\right]$ in logarithmic increments.

The second algorithm employed in this work is Support Vector Machines (SVM) [35]. SVM is currently used as the gold-standard algorithm for building infrared models where there is a non-linear relationship between infrared spectra and reference analysis [28,36]. This algorithm could be used for both regression analysis (referred to in this case as SVR) and for classification (SVC). In order to form the SVM model, SVRs will create either one or multiple hyperplanes in a high-dimensional space to make a regression of the data based on the dimension of the data. In order to model non-linear data with SVMs, different kernels (linear, RBF, polynomial and sigmoid) were used to calculate the output of the SVM. To ensure we have an optimal model to model the data using SVRs, the following three parameters were optimized or fine-tuned: C (the regularization parameter) (0.1, 1, 10, 100, 1000, 10,000), ϵ (the epsilon-insensitive parameter) (0.01, 0.1, 1, 10, 100) for regression only, and γ (the kernel parameter) (scale or auto).

The third algorithm employed in this work is the random forest (RF) [37]. This algorithm is employed in this work due to its ability to handle complex, high-dimensional spectral data without strict assumptions about data distribution and its inherent robustness to noise and outliers. Random forest has been used with milk mid-infrared spectra for classification [38,39] and regression [40]. Random Forest (RF) is an ensemble learning method based on a multitude of decision trees and widely used for both regression (RFR) and classification (RFC) models. RF can either average the individual trees to produce an overall prediction in regression, or use a plurality of votes to determine the overall classification. In either case, the number of trees (n_estimators: 100, 300 and 500), maximum depth of trees (max_depth: None, 10, 20) and the minimum number of samples per leaf (min_samples_leaf: 1, 2, 4) were the main hyperparameters that were tuned.

The last algorithm used in this work is the Gaussian Process (GP) [41]. This algorithm is chosen because GPR is well-regarded for its performance on small-to-medium-sized datasets and its handling of non-linear data such as overlapping peaks and non-linear relationships between spectral absorbance and chemical properties [42]. GPs produce a probabilistic prediction for both GPR and GPC (regression and classification); they output both a predicted value (the mean) and a measure of confidence (the variance). In essence, GPR assumes that any set of observations follows a multivariate Gaussian distribution. By using a kernel function which defines the similarity between different data points, the model can infer the value of a function at new, unseen points based on the observed data. In the Gaussian process algorithm, hyperparameters are parameters of the different kernel functions (constant kernel, RBF and white kernel) that control the behavior of the model.

Statistical metrics that were employed for both the regression and classification models were the Coefficient of Determination (R^2^) for measuring the amount of variance that can be explained by the independent variables, the Root Mean Squared Error (RMSE) for measuring the average error in prediction, and the Residual Predictive Deviation (RPD) for measuring the utility and stability of the model. For the classification models, overall accuracy was defined as the proportion of correct predictions, as well as Sensitivity and Specificity, which measure true positive and true negative rates, respectively, for each class. The threshold used to determine the two different classes of FFA was based on Kintner et al. [43].

A 10-fold cross-validation approach was employed on the calibration dataset to ensure that models were robust and to avoid the models being overtrained. To optimally determine the hyperparameters selected for each type of machine learning algorithm, the Grid Search method was systematically performed over a grid of specified hyperparameter combinations in order to identify the combination of hyperparameters that produces the lowest validation RMSE (for regression) or to maximize the scoring classification performance (for classification).

To evaluate model performance under realistic conditions, the dataset was initially partitioned randomly into an 80% calibration set and a 20% validation set. Model robustness was assessed using both cross-validation on the calibration data and an independent holdout test set using 20% of samples, ensuring that final performance metrics reflected unbiased predictive capabilities.

It is important to highlight that, when splitting the entire dataset, the induced-lipolysis dataset primarily consisted of unique biological samples subjected to specific resting times (0.5, 1, or 2 h) rather than repeated measures of the same sample. Verification was performed to ensure that no observations derived from the same original milk sample were simultaneously present in both the calibration and validation subsets, thereby preventing any information leakage.

To evaluate the impact of dataset composition and distribution shifts on predictive accuracy, model performance metrics ($R^{2}$ and RMSE) were also computed separately for the three main sample categories represented within the independent holdout test set: historical spontaneous lipolysis, induced lipolysis, and newly collected high-lipolysis field samples.

Data quality was maintained through a two-stage screening process designed to eliminate abnormal data. First, spectral outliers were addressed by calculating the standardized Mahalanobis distance (GH), which measures the multivariate distance of an individual spectrum from the mean spectrum of the dataset within a Principal Component Analysis (PCA) space [31]. For all samples within the calibration set, spectra with GH values exceeding 30 were removed (n = 2 records deleted). This relatively high threshold was intentionally selected to preserve spectral variability and increase the robustness of the developed models [31] while still filtering out genuine instrumentation or measurement anomalies.

Second, to develop the quantitative regression models, a reference-based filtering step was integrated directly into the cross-validation phase. This process was carried out separately for each algorithm and each target FFA. Initially, a calibration model was constructed using all available samples, followed by the calculation of prediction residuals. Samples with an absolute prediction residual exceeding 2.5 times the standard deviation (SD) of the residuals were classified as outliers and subsequently removed. A final model was then retrained on this refined calibration dataset. A standard deviation threshold of 2.5 was selected to reduce the likelihood of analytical errors and to detect potential sample misidentification. It is crucial to highlight that the outlier removal based on prediction errors was exclusively implemented in the regression models. This method was not utilized in the qualitative classification models, thereby guaranteeing that the classification algorithms were trained and assessed on the complete intricacy of the dataset.

Importantly, this outlier removal protocol was applied to individual FFA during calibration, while the holdout test set remained entirely unaltered to preserve the integrity of the final testing. The total proportion of data excluded through these two criteria was subsequently quantified and reported.

All data preprocessing, model training, and statistical analyses were performed using Python (version 3.12.3). The machine learning pipelines, hyperparameter tuning via Grid Search, and model evaluations were implemented using the Scikit-learn 1.8.0 [44] package.

## 3. Results and Discussion

### 3.1. Comparison of Free Fatty Acids Concentration with Two Reference Methods

**Figure 1:**
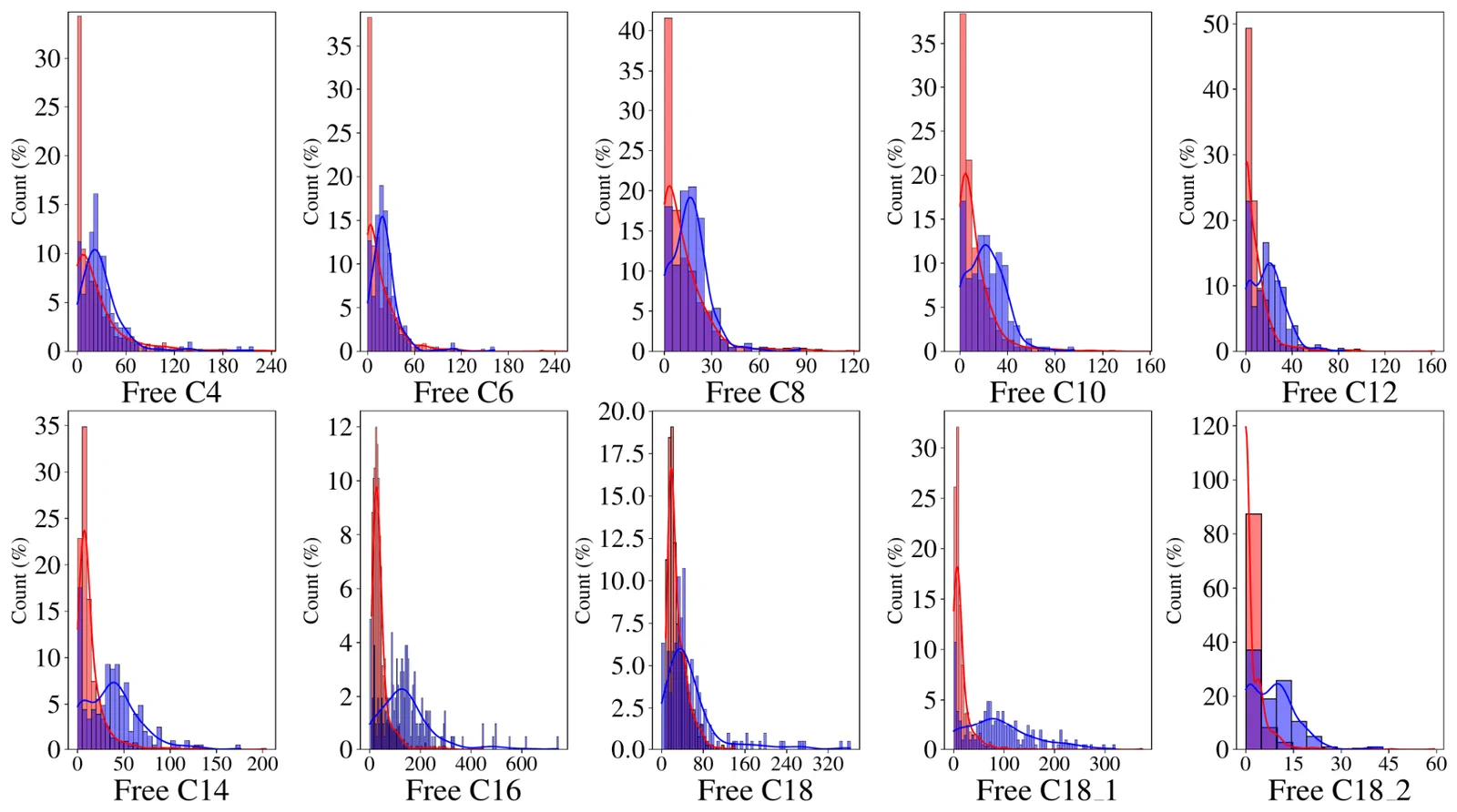
Distribution (count, in percent) of individual FFA in both datasets: in red, the method described by Christophe et al. [11] (n = 792); in blue, the method described in ISO 24191:2024|IDF 263:2024, adapted from Mannion et al. [20] (n = 248).

The distribution presented in Figure 1 and the descriptive statistics in Table 1 reveal several differences between the datasets. The spontaneous lipolysis and induced lipolysis datasets are those from Christophe et al. [11], whereas the selected lipolysis dataset was analyzed with the ISO 24191:2024|IDF 263:2024 method adapted from Mannion et al. [20]. The first observation was related to the difference in standard deviation (SD) for each FFA of the three distinct sample sets, with a higher SD for lab-induced lipolysis. However, the targeted high lipolysis samples showed a similar SD to the lab-induced lipolysis dataset. The second observation is the similar median for the lab-induced samples and the targeted high-lipolysis dataset, particularly for the SCFFA such as C4, C6 and C8, as well as C10 and C12. The last observation is a distinct shift in the distribution for the targeted high lipolysis dataset. Interestingly, we noted a marked displacement in the distribution of the higher-chain FFA, specifically C14, C16, and C18:1. This difference suggested a potential analytical effect associated with the reference methods and therefore warranted further investigation. To substantiate these results, it is essential to compare both methods using the same sample set.

To identify potential differences between the two reference methods, a set of 25 samples was analyzed with both quantification methods in order to elucidate the potential link between them.

Linear regression analyses were performed to quantify the agreement between the two reference methods, and the slope and bias are shown in Table 2. In order to obtain complementary information on the inter-comparability of the data, Bland-Altman, Deming and Passing-Bablok analyses were performed and are presented in Figure S1.

To evaluate the analytical performance of the relation between the two described methods, all fatty acids (from C4 to C18:2) and their respective groupings (SCFFA, MCFFA, LCFFA) were compared. Linearity criteria for this method are shown in Table 2 (i.e., slope, bias, and coefficient of determination (R^2^)).

**Figure 2:**
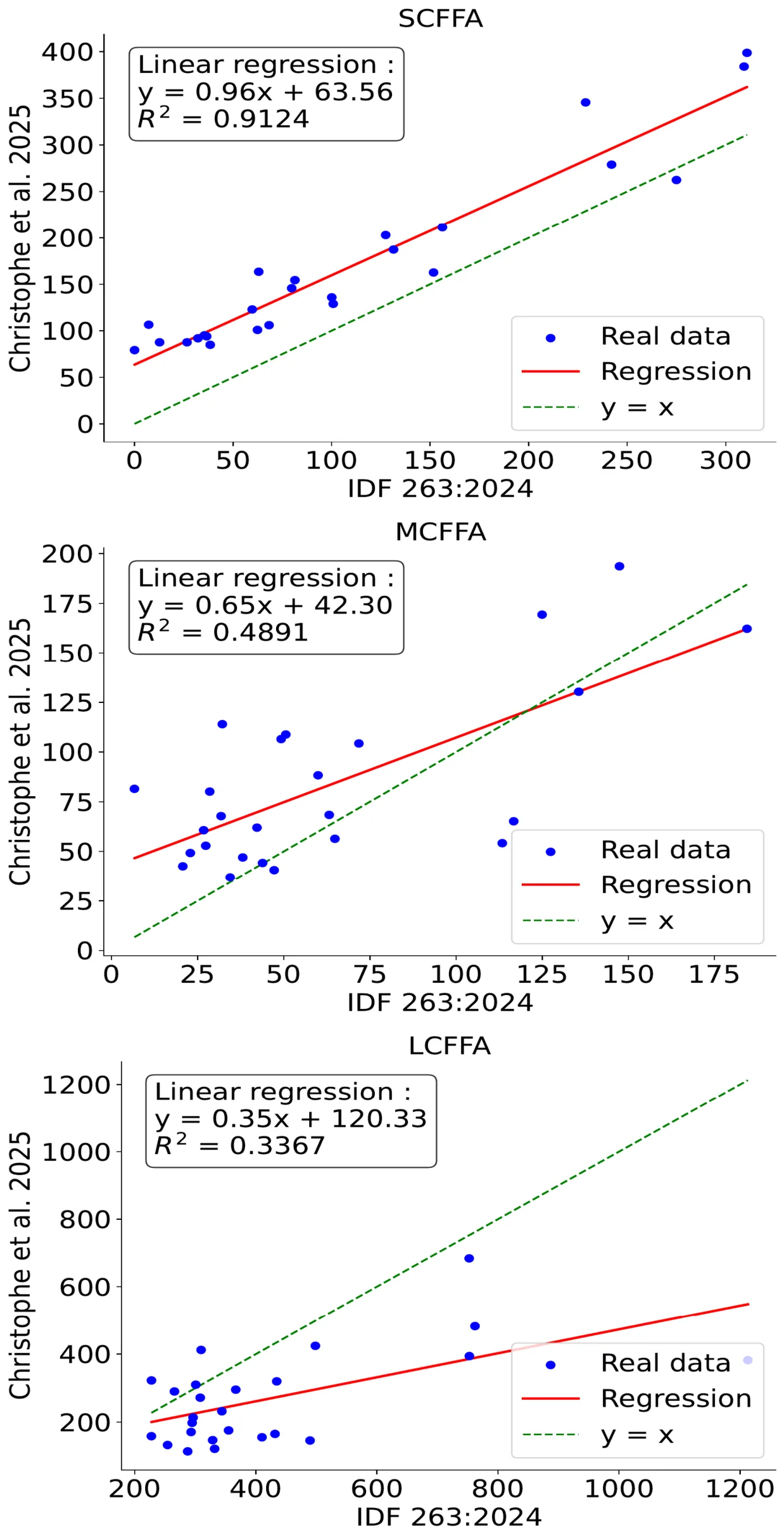
Comparison of 25 samples with a good distribution of grouped FFA obtained with the method described by Christophe et al. [11] and with the method described in ISO 24191:2024|IDF 263:2024, adapted from Mannion et al. [20].

Both methods have excellent predictive accuracy and excellent linearity when analyzing short-chain fatty acids (Figure 2). Both C4 and C6 performed exceptionally well (i.e., slopes were close to one (0.94 and 1.00, respectively) and both exhibited high values for R^2^ (0.91 and 0.93, respectively)). Furthermore, SCFFA had an overall slope of 0.96 and a high R^2^ of 0.91 (compared with C4 and C6 individually); however, the accumulated positive bias was 63.56 (Table 2).

As the carbon chain length increased, both linearity and accuracy progressively decreased. Of the last SCFFA compounds and the first MCFFA, C8 and C10 had acceptable slopes (0.92 and 0.82) and reasonably good correlations (R^2^ = 0.80 and 0.74). However, for the two other MCFFA, C12 and C14, the linearity statistics fell off sharply: slopes dropped to 0.61 and 0.65, while R2 values decreased to 0.38 and 0.53, respectively. Therefore, the total MCFFA group had moderate performance with a slope of only 0.65 and an R2 of 0.49.

The decrease in performance was even more marked within the long-chain free fatty acids (LCFFA) group, which had a low slope (0.35), a low correlation (0.34), and a large positive bias (120.33) (Table 2). Only C16 displayed a negative bias (−9.88) among all of the LCFFA components. Its slope was relatively good (0.75) but its R^2^ value was relatively low (0.47). There were also unsaturated long-chain fatty acids that showed poor slope and strong inaccuracies; C18:1 had low accuracy and linearity (slope = 0.52, R^2^ = 0.46) and C18:2 had a high slope (0.88) and very low correlation (R^2^ = 0.25).

These findings indicate that both methods agree closely for quantifying SCFFA, but their agreement diminishes for heavier, long-chain molecules. These results do not support merging datasets for MCFFA and LCFFA model development. Conversely, the strong agreement observed for SCFFA supported the integration of both datasets for subsequent model development.

The discrepancies between the two methods were most pronounced for LCFFA, with the ISO method consistently yielding higher concentrations. Several factors may contribute to these differences. First, the method derived from Mannion et al. [20] was specifically developed to maximize FFA recovery in complex dairy matrices through improved extraction and chromatographic procedures. Consequently, a greater recovery of LCFFA may be achieved compared with the methodology previously used by Christophe et al. [11]. In addition, LCFFA measurements may be more susceptible to low-level contamination originating from laboratory consumables or sample handling procedures. The protocol established by Christophe et al. [11] actively mitigates such artifacts by prioritizing glassware and implementing a hexane-washing step for plastic consumables. Previous studies have reported that plastic laboratory materials can release trace amounts of C16 and C18 fatty acids, potentially affecting quantitative measurements [45,46].

Alternative analytical parameters may provide an explanation for these observed discrepancies. Specifically, the derivatization procedures demonstrate substantial variation (for instance, the synthesis of butyl esters in contrast to MTBSTFA derivation). This procedural distinction modifies the volatility and thermal resilience of the LCFFA derivatives throughout gas chromatography analysis, frequently resulting in variable response factors. Additionally, the particular properties of the stationary phases used, as different columns were employed, determine the retention and elution characteristics of these macromolecular compounds. Collectively, these analytical inconsistencies establish an unreliable reference point for LCFFA within the consolidated datasets, thereby reinforcing the deliberate choice to confine the ultimate FT-MIR predictive models to the more analytically uniform SCFFA component.

Although the present study was not designed to identify the exact source of these discrepancies, the observed differences highlight the importance of carefully selecting and standardizing reference methods before combining datasets for calibration purposes.

### 3.2. FFA Model Composed of the Second Phase Dataset

Table 3 presents the calibration and cross-validation statistics for the quantification of the 248 samples of the second selection phase with the gold-standard PLS algorithm; each individual FFA (C4 to C18:1) was analyzed. The coefficients of determination (R^2^cal, R^2^cv), root mean square errors (RMSEcal, RMSEcv), and residual predictive deviation (RPDcal, RPDcv) were used to develop the models and to assess how accurate and reliable they were. Due to the restricted sample size and the experimental nature of this study, we opted against splitting the dataset into a holdout test set, choosing instead to utilize the full dataset for model development with no outlier elimination.

The models developed were found to be predictive with varying amounts of success; the model’s predicted success for SCFFA was greater than that of LCFFA. The best predictive performance was achieved for C4 and C6, where the cross-validation metrics for C4 (R^2^cv = 0.57) and C6 (R^2^cv = 0.56) and the RPDcv values for both FFA (C4 = 1.52; C6 = 1.51) were at or near the minimum acceptable predictive value of 1.5.

While the predictive models reported by Christophe et al. [11] outperformed those developed in the present study, this performance gap can be primarily attributed to differences in dataset volume, as their calibration database was three times larger than our current set. Furthermore, it should be emphasized that the data collection in this work was not originally intended to establish an independent, standalone model. Instead, the primary objective was to expand and enrich the existing dataset of Christophe et al. [11].

### 3.3. Merging of Datasets to Create a More Robust SCFFA Model Through Different Algorithms

To improve dataset robustness of Christophe et al. [11], the next step was to integrate real field samples exhibiting high lipolysis levels obtained in this study. The resulting dataset encompassed a broad range of values: low spontaneous lipolysis and variable (low to high) induced lipolysis from Christophe et al. [11], complemented by high spontaneous lipolysis values derived from the new field samples. Indeed, the complete dataset was composed of 313 spontaneous lipolysis samples, 479 lab-induced lipolysis samples and 248 spontaneous lipolysis samples selected for their high FFA content.

Mixing of the datasets was only possible for C4, C6, C8 and the sum of these FFA (SCFFA). Restricting model development to SCFFA was considered acceptable because these compounds are the primary contributors to lipolysis-related flavor defects.

The performance of modeling of the different SCFFA with five different algorithms is shown in Table 4.

Holdout test set results showed that model performance depended on the analyte being predicted, and no single algorithm consistently outperformed all others. While non-linear approaches improved the prediction of certain SCFFA, particularly C6 and total SCFFA, PLS remained highly competitive and provided the best performance for C4.

PLS regression was found to be the best C4 quantification method, generating the most accurate holdout test set results with an R^2^val of 0.84, and producing the lowest prediction error (RMSEval = 17.84); PLS also produced an outstanding residual predictive deviation (RPDval = 2.49). Although GPR attained similar performance (RPDval = 2.37), the linear model of PLS provided a modest competitive advantage for this highly volatile, short-chain molecule.

For the less volatile FFA C6, the application of non-linear regression modeling was clearly more beneficial than linear regression modeling. SVR produced the best validation metrics (R^2^val = 0.63, RPDval = 1.65), resulting in an increase from an unreliable screening metric to a reliable screening metric (RPD > 1.5), compared to the PLS baseline model (R^2^val = 0.53, RPDval = 1.46).

For the FFA C8, SVR produced the best validation metrics. SVR resulted in an R^2^val of 0.53 and RMSEval = 8.61 (RPDval = 1.46). When compared with PLS (RPDval = 1.36), SVR produced a clear improvement in terms of closeness to the acceptable screening threshold.

For the pooled SCFFA group, GPR provided the absolute best predictive performance. It maximized the correlation to an R^2^val of 0.80 and decreased the prediction error to RMSEval = 32.08, culminating in an excellent RPDval of 2.22. This marks a clear enhancement over PLS (RPDval = 2.11), suggesting that GPR is highly capable of capturing complex, aggregated matrix variances.

Random Forest (RF) suffers from classic ensemble overfitting in spectral chemometrics; its decision trees easily memorize the training set noise but fail to generalize to independent holdout test set samples [47].

The relative performance hierarchies (i.e., the ranking of the algorithms) and the predictive ability obtained for each SCFFA show a strong correspondence with those documented in previous studies [11]. According to published studies, the typical RPDval values reported are: 2.53 for C4, 1.57 for C6, and 1.46 for C8, while the mean RPDval for the combined SCFFA fraction is 2.10; these results are comparable to those reported for the overall performance of the reference models in our study with values of: 2.49 (C4), 1.65 (C6), 1.46 (C8), and 2.22 (SCFFA). However, it is important to note that there was considerable variation in the RMSEval of the models when they were used with holdout test set (i.e., measuring model accuracy). This increased RMSE can be attributed to the additional, high lipolyzed samples used to validate the models that were previously not in the same sample sets used to develop the model. The inclusion of a new type of lipolyzed samples into the validation dataset also increased the amount of matrix variability and chemical diversity present, thereby subjecting the models to a more realistic field situation than in previous studies, which typically used induced lipolyzed samples.

During the regression analysis, the performance of the various algorithms varied depending on the specific fatty acid being modeled. No single algorithm consistently outperformed all others across the different SCFFA. Model performance depended on the analyte considered, suggesting that the relationship between FT-MIR spectra and FFA concentrations differs according to fatty acid chain length and concentration range. RF models exhibit a significant decline in performance during cross-validation/validation. This decline is attributed to a common issue in spectral chemometrics: RF models are prone to overfitting, where decision trees effectively “memorize” noise in the training set but fail to generalize to independent validation samples [47,48].

Furthermore, when comparing computational efficiency, SVR proved disadvantageous; it requires high computational resources without providing a proportional improvement in performance. In contrast, KRR and GPR offered a more favorable balance, as they require less computational effort while simultaneously enhancing model accuracy.

A sensitivity analysis using the Passing-Bablok correction was performed on the independent holdout test set using the PLS algorithm for the SCFFA model. The impact of this mathematical correction is detailed in Supplementary Table S1. While the correction maintained similar cross-validation metrics, it resulted in decreased predictive performance during external validation. Consequently, the final combined models did not incorporate this correction. We hypothesize that embedding this inherent “multi-method variability” into the calibration space actively benefits the model’s practical utility. As a high-throughput screening tool designed for the dairy sector, the models will inevitably encounter samples analyzed by various laboratories employing slightly different internal protocols. Exposing the machine learning algorithms to this methodological variation during training prevents overfitting to a single laboratory’s strict analytical baseline, thereby enhancing the global robustness and generalizability of the models for routine screening applications.

A stratified analysis of the test set offers additional insights into the distribution of prediction errors among various sample populations (Supplementary Table S2). As anticipated, the historical spontaneous lipolysis group demonstrated the lowest absolute prediction errors (e.g., RMSE of 19.82 mg/L for SCFFA), indicative of its narrower and lower concentration range. Conversely, the increased prediction errors previously mentioned are evidently linked to the wider concentration distributions of the induced-lipolysis and newly introduced high-lipolysis field samples, which resulted in higher RMSEs for SCFFA (42.27 mg/L and 38.09 mg/L, respectively). Given that RMSE is particularly sensitive to the scale of the target variable, it is mathematically expected that higher errors will occur at elevated concentrations. Despite this rise in absolute error, the incorporation of the natural high-lipolysis field samples proved to be significantly advantageous for model stability: it preserved a consistent $R^{2}$ for SCFFA (>0.72 across all subsets) and markedly enhanced the explained variance for C6 ($R^{2}=0.67$) and C8 ($R^{2}=0.61$) in comparison to the mechanically induced samples. This validates that augmenting the calibration set with naturally lipolyzed extreme samples effectively broadens the model’s operational range without compromising its relative predictive capability.

While the combined dataset exhibited a higher $R^{2}$, it is important to note that this increase is primarily driven by the broader concentration range and wider data distribution introduced by the new field samples, rather than an absolute improvement in algorithmic precision. Therefore, these subgroup results do not necessarily demonstrate that the inclusion of the new samples improved the model itself. Instead, they demonstrate the model’s reasonably consistent predictive performance and robustness across distinct sample categories albeit with varying absolute errors that are inherently linked to their specific concentration ranges.

### 3.4. Classification Models of SCFFA Through Different Algorithms

The classification performance of five machine learning algorithms (PLS-DA, kernel ridge classification (KRC), support vector machine classification (SVC), random forest classification (RFC) and Gaussian process classification (GPC)) was evaluated across three distinct FFA and the sum of these FFA (C4, C6, C8, and SCFFA) based on specific concentration thresholds [43] summarized in Table 5.

Across all target analytes, PLS-DA, KRC, and GPC consistently emerged as the top-performing models, generally yielding the highest classification accuracies (ranging from 82% to 87%). Conversely, as in the quantification modeling, RFC demonstrated the lowest performance, underperforming in accuracy, specificity, and sensitivity for nearly every FFA in validation. This suggests that tree-based ensemble methods may struggle with the high-dimensional or highly correlated nature of the spectral data compared to kernel-based or linear discriminant approaches.

For C4 (threshold: 27.5 mg/L), PLS-DA achieved the highest accuracy (84%) and specificity (94%). However, all models exhibited a notable drop in sensitivity for this analyte, with RFC dropping as low as 43% and PLS-DA peaking at only 63%. This pronounced imbalance implies that while the models are highly reliable at identifying true negatives (samples below the threshold), they struggle to accurately detect positive samples.

The classification of C6 (16 mg/L) and C8 (8.3 mg/L) showed a much better balance between sensitivity and specificity. For C6, GPC achieved the highest accuracy (87%), closely followed by KRC and SVC (86%). Interestingly, for C8, the trend inverted compared to C4: all models showed higher sensitivity (up to 90% for KRC) than specificity (72–82%), indicating a superior capacity for detecting positive samples.

When classifying the cumulative SCFFA content (threshold: 51.8 mg/L), PLS-DA delivered the highest overall accuracy (85%) and specificity (90%), while maintaining a robust sensitivity of 78%. KRC and GPC performed comparably, each achieving 84% accuracy, further reinforcing the suitability of kernel-based architectures for this application.

However, the relevance of this specific threshold may be questioned, given that it was established nearly 60 years ago. Since then, analytical quantification methods have advanced significantly and the perception of rancid flavor may also have changed, which potentially explains the observed limitations in predicting C4 concentrations. This threshold serves as a purely chemical indicator. The absence of a sensory panel in this study means that the models focus on the exceedance of historical chemical limits rather than directly assessing perceived sensory rancidity. In light of the changes in dairy cattle genetics, feeding practices, and milk matrices over the years, a significant direction for future research would be to carry out extensive sensory evaluations to either validate or adjust these concentration thresholds for current dairy populations.

Nevertheless, from a practical point of view, these qualitative models could serve as an effective screening tool to detect milk samples with elevated SCFFA concentrations. This would allow for the rapid identification and exclusion of individual milk samples at risk of sensory defects, thereby preventing the contamination of bulk tank milk.

In practice, predicting SCFFA, the primary drivers of sensory defects in milk, with an 85% classification accuracy provides significant operational benefits. At the dairy processing level, where mechanical handling of raw milk frequently induces lipolysis, this model enables the selective exclusion of affected batches, thereby preventing bulk lot contamination. At the individual cow level, the increased milking frequency associated with automatic milking systems (AMS) tends to elevate lipolysis rates [8,49]; farmers could thus directly segregate milk exceeding the SCFFA threshold. Finally, the model’s robustness, achieved through the inclusion of three distinct datasets representing diverse lipolysis mechanisms (spontaneous, lab-induced, and targeted high-lipolysis samples), supports its application as a versatile proxy across various field scenarios to detect and prevent flavor defects.

Finally, despite the overall robustness of the models, the merging of reference values obtained from two distinct analytical methods constitutes a recognized limitation of this study. As indicated by the comparative analyses, a systematic difference exists between the historical method and the ISO 24191:2024|IDF 263:2024 standard, even for the SCFFA fraction. Therefore, while the current models serve as effective and necessary high-throughput screening tools, this methodological bias further highlights the need for future studies to establish updated, method-specific thresholds validated by modern sensory panels.

## 4. Conclusions

In conclusion, the inclusion of samples with high spontaneous lipolysis successfully expanded the variability of the dataset. Furthermore, it was demonstrated that altering the FFA determination method directly impacted the quantification of MCFFA and LCFFA. Consequently, the final models focused on developing prediction equations for SCFFA, which are primarily responsible for flavor defects in milk. This work demonstrates that while incorporating new samples with high lipolysis maintained comparable predictive performance and enhanced model robustness, it inherently increased the prediction error. Several machine learning algorithms were evaluated in this study. Model performance depended on the FFA considered, and no single algorithm consistently outperformed all others. While PLS remained highly competitive, non-linear approaches provided analyte-specific improvements, particularly for C6 and total SCFFA prediction.

Finally, the best-performing classification models achieved an accuracy between 84% and 87% in identifying samples that exceeded the historical chemical threshold associated with rancidity flavor. In practice, these qualitative models may therefore serve as an effective screening tool for early detection of milk samples at risk of lipolysis-related flavor defects, enabling the identification and exclusion of defective individual milk samples to prevent the contamination of bulk tank milk.

## Figures and Tables

**Table 1:** Descriptive statistics of the different datasets for each FFA in mg/L.

**Spontaneous Lipolysis Dataset [11]**						
**FFA**	**Min**	**Med.**	**Max**	**Mean**	**SD**	**CV (%)**
C4	2.0	2.2	40.2	4.2	5.0	119
C6	3.5	3.5	30.7	4.4	3.1	70
C8	2.5	2.5	19.5	3.3	2.1	64
C10	3.5	3.6	28.1	5.1	3.1	62
C12	5.5	5.5	21.2	6.1	1.8	29
C14	3.5	7.1	47.8	8.1	5.3	66
C16	7.5	25.6	105.8	27.4	15.6	57
C18	7.5	25.5	93.0	29.6	15.9	54
C18:1	4.5	7.2	211.5	12.7	18.1	142
C18:2	3.0	3.0	22.9	3.6	2.0	55
SCFFA	8.0	8.4	90.4	12.0	10.0	83
MCFFA	12.5	16.5	87.3	19.2	9.5	49
LCFFA	22.5	66.3	354.3	73.3	44.6	61
**Induced Lipolysis Dataset [11]**						
**FFA**	**Min**	**Med.**	**Max**	**Mean**	**SD**	**CV (%)**
C4	2.0	26.2	638.7	42.0	51.5	122
C6	3.5	20.7	395.8	27.9	30.2	108
C8	2.5	14.7	225.3	19.2	20.5	107
C10	3.5	13.6	325.9	19.4	23.7	122
C12	5.5	8.4	162.6	13.8	15.2	110
C14	3.5	10.7	203.7	18.3	21.3	117
C16	9.1	34.3	445.8	49.9	48.2	97
C18	8.2	21.8	140.0	29.7	21.4	72
C18:1	4.5	9.1	374.2	17.8	28.7	161
C18:2	3.0	3.0	59.3	4.4	4.5	103
SCFFA	8.0	64.3	1259.7	89.1	96.5	108
MCFFA	12.5	33.6	692.2	51.5	57.6	112
LCFFA	25.1	72.5	771.1	101.8	92.2	91
**Targeted High Lipolysis Dataset**						
**FFA**	**Min**	**Med.**	**Max**	**Mean**	**SD**	**CV (%)**
C4	0	24.3	217.4	33.0	32.8	99
C6	0	19.7	162.4	22.8	20.2	88
C8	0	14.1	86.0	14.5	12.1	84
C10	0	19.0	95.7	19.8	16.0	81
C12	0	15.9	84.6	16.1	14.8	92
C14	0	34.6	176.0	36.7	28.6	78
C16	0	137.3	743.5	148.5	107.9	73
C18	0	41.5	362.4	54.1	52.7	97
C18:1	0	76.4	500.2	89.1	69.9	78
C18:2	0	6.0	43.1	7.0	7.6	109
SCFFA	0	59.1	446.7	70.3	62.3	89
MCFFA	0	64.9	356.3	72.6	57.4	79
LCFFA	0	269.4	1319.3	297.8	213.9	72

**Table 2:** Slope, bias and R^2^ of the comparison between both chromatographic methods.

FFA	Slope	Bias (mg/L)	$R^{2}$
C4	0.94	30.08	0.91
C6	1.00	17.73	0.93
C8	0.92	16.11	0.80
C10	0.82	16.82	0.74
C12	0.61	13.71	0.38
C14	0.65	11.24	0.53
C16	0.75	−9.88	0.47
C18	0.73	7.35	0.59
C18:1	0.52	23.30	0.46
C18:2	0.88	13.31	0.25
SCFFA	0.96	63.56	0.91
MCFFA	0.65	42.30	0.49
LCFFA	0.35	120.33	0.34

**Table 3:** Performance of modeling based on the ISO reference dataset.

FFA	R^2^cal	R^2^cv	RMSEcal	RMSEcv	RPDcal	RPDcv
C4	0.73	0.57	12.53	15.91	1.93	1.52
C6	0.64	0.56	8.74	9.7	1.68	1.51
C8	0.54	0.46	6.92	7.54	1.48	1.36
C10	0.51	0.38	9.77	10.96	1.43	1.27
C12	0.47	0.37	9.17	10.04	1.37	1.26
C14	0.45	0.26	18.54	21.46	1.34	1.17
C16	0.52	0.38	55.48	62.5	1.43	1.27
C18	0.46	0.28	24.17	28.0	1.36	1.18
C18:1	0.38	0.26	51.94	57.05	1.27	1.16

**Table 4:** Performance of quantification of SCFFA models with different algorithms on a 10-fold cross-validation and a random holdout test set. A total of 1040 samples, of which 832 are in the calibration set and 208 in the holdout test set. Values in brackets indicate the number of outliers excluded from the models.

FFA	Metric	PLS	KRR	SVR	RF	GPR
**C4** (10)	R^2^cv	0.71	0.73	0.67	0.53	0.73
	R^2^val	0.84	0.81	0.70	0.50	0.82
	RMSEcv	16.72	16.39	16.52	18.81	16.00
	RMSEval	17.84	19.14	24.28	31.29	18.68
	RPDcv	1.87	1.91	1.73	1.45	1.92
	RPDval	2.49	2.32	1.83	1.42	2.37
**C6** (12)	R^2^cv	0.69	0.70	0.68	0.48	0.69
	R^2^val	0.53	0.56	0.63	0.48	0.60
	RMSEcv	11.18	10.95	10.29	11.94	11.23
	RMSEval	13.37	12.86	11.79	14.06	12.40
	RPDcv	1.79	1.82	1.77	1.39	1.79
	RPDval	1.46	1.52	1.65	1.39	1.57
**C8** (16)	R^2^cv	0.62	0.64	0.64	0.46	0.70
	R^2^val	0.46	0.50	0.53	0.42	0.47
	RMSEcv	7.88	7.30	7.22	8.09	6.93
	RMSEval	9.24	8.92	8.61	9.58	9.14
	RPDcv	1.63	1.68	1.68	1.36	1.81
	RPDval	1.36	1.41	1.46	1.32	1.38
**SCFFA** (9)	R^2^cv	0.69	0.71	0.66	0.50	0.72
	R^2^val	0.77	0.79	0.72	0.53	0.80
	RMSEcv	36.78	34.29	32.40	36.83	34.42
	RMSEval	33.81	32.83	37.47	48.68	32.08
	RPDcv	1.80	1.85	1.72	1.42	1.89
	RPDval	2.11	2.17	1.90	1.46	2.22

**Table 5:** Performance of classification SCFFA models (i.e., accuracy, specificity and sensitivity) with different algorithms (PLS-DA, KRC, SVC, RFC and GPC) on the 208 samples of the holdout test set.

FFA	Threshold	(mg/L)	PLS-DA	KRC	SVC	RFC	GPC
C4	27.5	Accuracy	84%	82%	80%	75%	83%
		Specificity	94%	92%	91%	89%	93%
		Sensitivity	63%	61%	55%	43%	61%
C6	16	Accuracy	84%	86%	86%	80%	87%
		Specificity	88%	88%	87%	89%	90%
		Sensitivity	78%	83%	84%	69%	81%
C8	8.3	Accuracy	82%	83%	85%	79%	84%
		Specificity	76%	76%	82%	72%	80%
		Sensitivity	88%	90%	89%	87%	88%
SCFFA	51.8	Accuracy	85%	84%	82%	77%	84%
		Specificity	90%	87%	84%	86%	88%
		Sensitivity	78%	78%	78%	65%	77%

## Data Availability

The original contributions presented in the study are included in the article/Supplementary Materials, further inquiries can be directed to the corresponding authors.

## Supplementary Materials

The following supporting information can be downloaded at: https://www.mdpi.com/article/10.3390/foods15193423/s1, Figure S1. Comparison using Bland-Altman, Deming and Passing-Bablock regression of 25 samples with a good distribution of grouped FFA coming from the method described by Christophe et al. 2025 [11] and the methods described by ISO/IDF 263:2024 adapted from the methods of Mannion et al. [20]; Table S1. R^2^, RMSE and RPD of validation of PLS models on SCFFA without (A) and with (B) Passing-Bablok correction; Table S2. R^2^ and RMSE of validation grouped by samples from the induced lipolysis dataset, the spontaneous lipolysis dataset and the high-lipolysis field dataset.

## Abbreviations

The following abbreviations are used in this manuscript:

FT-MIR	Fourier-transform mid-infrared
FFA	Free Fatty Acids
SCFFA	Short-Chain Free Fatty Acids
MCFFA	Medium-Chain Free Fatty Acids
LCFFA	Long-Chain Free Fatty Acids
PLS	Partial Least Squares
KRR	Kernel Ridge Regression
SVR	Support Vector Regression
RF	Random Forest
GPR	Gaussian Process Regression
AMS	Automatic Milking System
AWE	Association Wallonne de l’Élevage
BDI	Bureau of Dairy Industries
CDL	Comité du Lait
CRA-W	Walloon Agricultural Research Centre
DHI	Dairy Herd Improvement
EMR	European Milk Recording
FABE	Fatty Acid Butyl Ester
GH	Standardized Mahalanobis Distance
GP	Gaussian Process
GPC	Gaussian Process Classification
ICAR	International Committee for Animal Recording
KRC	Kernel Ridge Classification
LPL	Lipoprotein Lipase
MFGM	Milk Fat Globule Membrane
MTBSTFA	N-Methyl-N-(tert-butyldimethylsilyl)trifluoroacetamide
PLS-DA	Partial Least Squares Discriminant Analysis
PLSR	Partial Least Squares Regression
RFC	Random Forest Classification
RFR	Random Forest Regression
SVC	Support Vector Classification
SVM	Support Vector Machine
TAG	Triacylglycerol
