# Supplementary material for: Enhancing FT-MIR Prediction of Short-Chain Free Fatty Acids in Milk Through High-Lipolysis Field Samples, Reference Method Evaluation and Machine Learning Approaches
Source: Foods. 2026 Sep 24;15(19):3423. doi: 10.3390/foods15193423 (PMC13664496; doi:10.3390/foods15193423)
Supplement: Supplementary file 1 [file foods-15-03423-s001.zip › foods-4523910-supplementary.pdf]

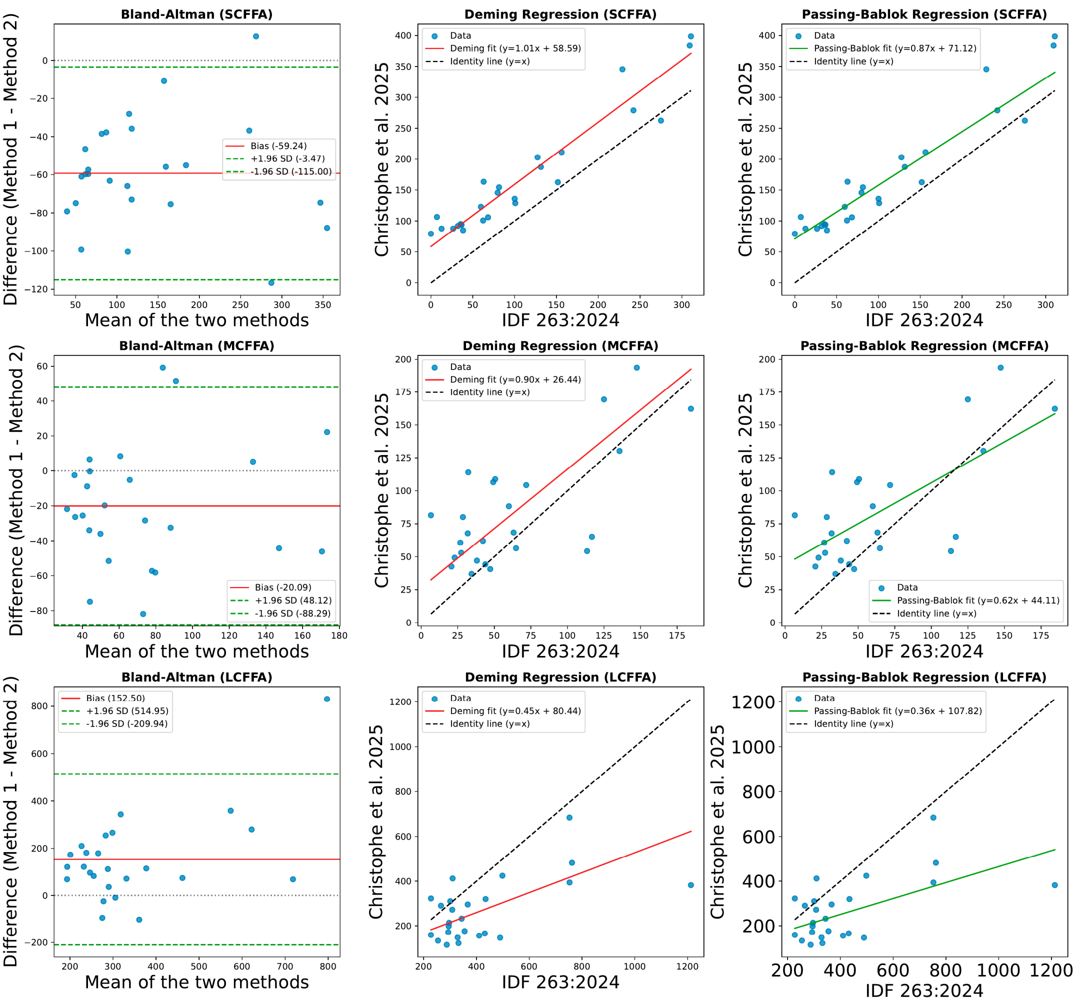

Figure S1. Comparison of 25 samples with a good distribution of grouped FFA coming from the method described by Christophe et al. 2025 and the methods described by ISO/IDF 263:2024 adapted from the methods of Mannion et al [20]

Table S1. R^2, RMSE and RPD of validation of PLS models on SCFFA without (A) and with (B) Passing-Balock corection.

	**A**	**B**
R^2_{CV}	0.69	0.69
R^2_{val}	0.77	0.74
$RMSE_{CV}$	36.78	36.12
$RMSE_{val}$	33.81	44.48
RPD_{CV}	1.8	1.80
RPD_{val}	2.11	1.96

Table S2. R^2 and RMSE of validation group by samples from Induced Lipolysis Dataset, Spontaneous lipolysis dataset and High lipolysis Field Dataset.

	**Induced Lipolysis**		**Spontanneous lipolysis**		**High lipolysis Field**	
FFA	R^2	RMSE	R^2	RMSE	R^2	RMSE
C4	0.83	22.80	0.87	7.99	0.71	21.57
C6	0.28	17.25	0.47	8.65	0.67	12.21
C8	0.17	12.61	0.50	5.19	0.61	7.20
SCFFA	0.74	42.27	0.74	19.82	0.72	38.09
